# Awareness of Metabolic Bariatric Surgery Among Medical Students and House Officers in Lagos, Nigeria: A Cross-Sectional Online Survey

**DOI:** 10.7759/cureus.77126

**Published:** 2025-01-08

**Authors:** Tioluwanimi Ojeola, Joshua Olajugba, Oluwatobi Shekoni, Oluwabusayo Aluko, Emeka Nwadinigwe

**Affiliations:** 1 General Surgery, Northern Ireland Medical and Dental Training Agency, Belfast, GBR; 2 Surgery, Surgery Interest Group, Africa (SIGAf), Lagos, NGA; 3 Vascular Surgery, Sheffield Teaching Hospitals NHS Foundation Trust, Sheffield, GBR; 4 Trauma and Orthopaedics, University Hospital Southampton NHS Foundation Trust, Southampton, GBR

**Keywords:** bariatric surgery, final-year medical students, house officer, lagos, nigeria, obesity

## Abstract

Background

Obesity poses a significant public health challenge globally as it is a risk factor for many non-communicable diseases (NCDs). Metabolic bariatric surgery (MBS) has proven to be a reliable method for ensuring sustained weight loss and sometimes resolution of obesity-related diseases. However, its uptake and utilization in Nigeria are low due to a variety of reasons, one of which may be insufficient knowledge and exposure to surgical methods of weight loss in medical school curricula. This study evaluated knowledge and attitudes regarding MBS among final-year medical students and house officers (HOs) in Lagos, Nigeria, who represent primary sources of health information for patients.

Methods

A cross-sectional study was conducted using a 19-point structured online questionnaire, which was divided into the following sections: demographics, knowledge assessment, and attitude measurement. This was distributed electronically using a convenience sampling technique to final-year medical students and HOs across Lagos teaching hospitals (April-July 2024). Ethical approval was not required for this study. Data analysis was performed using SPSS v27.0.1 (IBM Corp., Armonk, NY).

Results

A total of 203 responses were received. Of these, 117 respondents were female (57.6%), and 86 were male (42.4%). Final-year medical students accounted for 130 (64%), while HOs made up the remaining 73 (36%). Only eight respondents (3.9%) correctly identified all three primary indications for MBS. The failure of conservative methods of weight loss was the most correctly identified indication for MBS, selected by 66% of participants (n=134). Recognition of gastric bypass and sleeve gastrectomy was moderate, with 66.5% (n=135) and 63.5% (n=129) of respondents aware of these methods, respectively. However, only 29.6% (n=60) of respondents were aware of endoscopic balloon insertion. Notably, 94.6% (n=192) were unaware of bariatric services at their institutions, and 41.9% (n=85) reported no exposure to bariatric surgery in their medical school curriculum. There was significant uncertainty regarding surgical risk, with 47.3% (n=96) unable to compare MBS risks to common abdominal procedures. Additionally, 47.3% (96) anticipated social stigma associated with surgical weight loss interventions.

Conclusion

Nigeria is confronting a growing obesity epidemic that underscores the urgent need for a comprehensive national strategy. Central to this approach is the enhancement of medical education on obesity and MBS through targeted curriculum updates, ongoing professional development programs, and specialized training workshops. Such measures are essential to improving patient care and addressing the escalating burden of obesity in Nigeria.

## Introduction

Obesity is a global pandemic, and its incidence is estimated to be increasing even in low- and middle-income countries like Nigeria, where rising income, urban migration, and expanding access to processed foods are seen as key drivers [1,2]. A recent review found that 46.8 million people in Nigeria suffer from central obesity, with roughly 54% of them being female [3]. The prevalence of central obesity was more than twice as high in the southern regions as in the northern ones (48% vs. 18%) [3].

Obesity is well established as a risk factor for many non-communicable diseases (NCDs), with type 2 diabetes having the strongest association with obesity. Osteoarthritis, gastrointestinal disorders, gastroesophageal reflux disease, cholelithiasis, non-alcoholic fatty liver disease (NAFLD), endometrial breast cancer, coronary artery disease, stroke, venous stasis, deep vein thrombosis, hypertension, and colorectal cancer are additional conditions linked to obesity [2,4]. After smoking, obesity is the second-largest cause of cancer [4], all of which result in premature death, diminished quality of life, and disability, consequently increasing healthcare-related costs and adding to the already burdened health system [5], and is therefore rightly considered a condition of public health significance.

The most common method of defining obesity is the body mass index (BMI), measured in kg/m², with a BMI of 30 kg/m² or higher classified as obesity. Life expectancy is lowered by two to four years for those with a BMI of 30-35 and by 10 years for those with a BMI greater than 40 [6]. Research suggests that people who have access to obesity treatment are more inclined to try to lose weight [7]. Unfortunately, for many obese people, this is not the case, as they do not receive a diagnosis of obesity and accompanying care [8]. 

Apart from the health benefits, there are also societal and beauty implications of losing weight, resulting in a multi-billion-dollar industry offering products (the effectiveness of some, however, questionable) to achieve this goal [9]. The cornerstones of managing obesity include exercise, dietary adjustments, and cognitive behavioral strategies [10]. However, for certain patients, metabolic bariatric surgery (MBS) or medication is necessary. MBS, a surgical method of limiting the amount of nutrients consumed or absorbed, has been proven to be effective in achieving weight loss significant enough to cure or ameliorate the severity of some obesity-associated illnesses [4,5]. Notably, significant improvements in glycemic control often occur independently of and precede substantial weight loss. This early optimization of blood sugar levels suggests the involvement of complex processes beyond weight reduction, including changes in gut hormone levels and modifications in metabolic signaling pathways [11].

It is, however, still an area of limited exposure and practice among surgeons and other medical professionals in Nigeria, as its implementation faces a multitude of challenges, including limited expertise and training opportunities, lack of multidisciplinary teams, absence of national obesity protocols, high costs, slow adoption of minimally invasive surgical techniques, low public awareness, and suboptimal referral practices from peripheral hospitals [5]. This article aims to assess the knowledge and attitudes of final-year medical students and house officers (HOs) in Lagos, Nigeria, toward MBS. This study population represents the frontline of the health workforce in Nigeria, who will likely be responsible for educating and/or referring patients who need MBS. Part of this project was presented as a poster at the INCISION UK Global Surgery and Research Conference in November 2024.

## Materials and methods

We carried out a cross-sectional observational study among HOs and final-year medical students in Lagos, Nigeria using a 19-point structured questionnaire modified from previous similar studies ​[12,13]​. Lagos is the most populous state in Nigeria [14] and has a good mix of federal- and state-funded teaching hospitals, as well as general hospitals offering housemanship training opportunities at secondary and tertiary care levels. This study population represents the frontline of the health workforce in Nigeria, who will most likely be responsible for educating patients inquiring about the topic and/or referring patients who need MBS. They also are more likely to provide more accurate replies on the inclusion of MBS in their medical school curriculum. 

The questionnaire was divided into the following sections: demographics, knowledge assessment, and attitude measurement. The demographic section recorded information about sex, professional level (final-year medical student or HO), medical school, and institution where the HOs work. The knowledge section contained questions testing knowledge of the indications, complications, and mechanisms of action of MBS. The attitude section assessed respondents' opinions on safety risks, the likelihood of referral, confidence in providing information to patients, etc. A copy of the questionnaire used is available in the appendix section. 

We conducted a pilot study in which the survey was sent to the 500-level students of the College of Medicine, University of Lagos. Qualitative feedback was obtained on the clarity of the questions, and adjustments were made to the phrasing as appropriate.

This questionnaire was converted to a Google Form (Google LLC, Mountain View, CA, USA) and distributed using a convenience sampling technique via the internet to various WhatsApp chat groups containing final-year medical students and HOs across teaching and general hospitals in Lagos, Nigeria, between April and July 2024.

All participants provided informed consent after receiving a detailed explanation of the study's purpose and objectives. They were explicitly informed of their right to withdraw from the research at any point without facing any negative consequences. The study implemented comprehensive confidentiality measures to ensure that all data collected would be used solely for research purposes and that individual identities would remain strictly anonymous. 

Responses were analyzed using SPSS v27.0.1 (IBM Corp., Armonk, NY). The frequency of events is presented as frequencies and percentages, with testing performed using chi-square or Fisher’s exact test where applicable. P-values of <0.05 were considered statistically significant.

## Results

Demographics

Responses were received from 203 participants. Of these, 57.6% were female (n=117) and 42.4% were male (n=86). Sixty-four percent (n=130) of respondents were final-year medical students, while 36% (n=73) were house officers (HOs). Additionally, 78.3% (n=159) of respondents were either current students or graduates of the College of Medicine, University of Lagos. Among the HOs, 74% (n=54) were working at the Lagos University Teaching Hospital (LUTH).

Knowledge

Eighty percent of participants (n=163) correctly identified metabolic medicine as the aspect of medicine concerned with the treatment of obesity. The failure of conservative methods of weight loss was the most correctly identified indication for MBS, selected by 134 (66%) participants. Thirty-three point five percent (n=68) selected BMI >40 kg/m², and 31% (n=63) selected the presence of comorbid conditions of obesity with BMI >35 kg/m² as indications for MBS. Only 3.9% (n=8) selected all three indications correctly, and 45.3% (n=92) selected no correct indications based on the previous NIH Consensus in 1991 [15].

A total of 136 participants (67%) correctly identified the effect of MBS as limiting the ability to consume, digest, or absorb nutrients through surgical operations on the GI tract, with HOs being more likely to select the correct option (p=0.012). Seventy percent of respondents (n=142) chose laparoscopic surgery as the safest technique.

Gastric bypass and sleeve gastrectomy were the most correctly identified MBS procedures, selected by 66.5% (n=135) and 63.5% (n=129) of respondents, respectively. Endoscopic balloon insertion was selected by only 29.6% (n=60) of respondents. Additionally, 78.8% (n=156) identified nutrient malabsorption as a common complication of bariatric procedures.

Attitude

Forty-one point nine percent (n=85) reported that there was no mention of MBS in their medical school curriculum, and only 2% (n=4) said they were given sufficient information about MBS while in medical school.

Almost all participants, 94.6% (n=192), are unaware that bariatric surgical procedures are offered at their current teaching hospitals, with only 37.9% (n=77) indicating they are aware of bariatric procedures being performed in Nigeria. Additionally, 18.7% (n=38) believe bariatric procedures are riskier than other common abdominal surgeries, while 47.3% (n=96) are unsure if there is an increased risk, as demonstrated in Table 1.

**Table 1:** Summary of attitude questions presented in the Likert scale

Question	Strongly Disagree (%)	Disagree (%)	Undecided (%)	Agree (%)	Strongly Agree (%)	Total (%)
I feel bariatric surgery is a useful tool for treating obesity	7 (3.4)	12 (5.9)	71 (35.0)	57 (28.1)	56 (27.6)	203 (100)
I feel bariatric surgery is a safe option for treating obesity	7 (3.4)	25 (12.3)	103 (50.7)	42 (20.7)	26 (12.8)	203 (100)
If a patient meets the standard criteria for bariatric surgery, I would recommend evaluation by a bariatric surgeon	15 (2.5)	7 (3.4)	56 (27.6)	60 (29.6)	75 (36.9)	203 (100)
If a patient asks me about surgical options for weight loss, I feel confident to provide a satisfactory answer to them	32 (15.8)	44 (21.7)	67 (33.0)	36 (17.7)	24 (11.8)	203 (100)

Almost half of the respondents (47.3% (n=96)) think there is or will be a social stigma associated with weight loss using surgical methods. 27.6% (n=56) and 28.1% (n=57) strongly agree and agree, respectively, that MBS is a useful tool for treating obesity. However, about half (50.7% (n=103)) are ambivalent about its safety, and 33.5% (n=68) agree or strongly agree that it poses a safe option for obesity treatment. Most participants (66.5% (n=135)) would refer patients meeting the criteria to a bariatric surgeon. Additionally, 37.5% (n=76) were not confident in providing information to patients about the surgical management of obesity.

## Discussion

Despite widespread poverty, the incidence of obesity and its associated illnesses is on the rise in Nigeria with the increasing inclusion of high-fat and high-sugar processed foods, often seen as symbols of wealth and status, in everyday diets [5]. Like the four horsemen of the apocalypse, obesity is associated with cardiovascular disease, type 2 diabetes, and cancer, diseases that wreak havoc on human bodies and populations. As such effective treatment of obesity is a necessity for building healthy societies [4,5].

Although dietary modification and exercise had previously been the mainstay of treatment given to high BMI individuals, modern pharmacological agents like semaglutide have now been shown to cause clinically significant weight loss up to 9.4-16% of body weight [16]. They, however, have the downside of a high risk of regaining lost weight if the injections are stopped. MBS, however, is currently the sole therapeutic intervention capable of achieving significant and sustained weight loss in individuals with obesity with patients maintaining an average weight loss of 25% of their initial body weight five years after their surgery [17]. Its effects are mediated by complex interactions and changes in gastrointestinal hormones like Ghrelin, glucagon-like peptide-1, and peptide YY [18]. MBS also has the added advantage of total or near complete resolution of obesity-associated comorbidities with diabetic patients, for example, exhibiting a superior rate of glycemic regulation and lower glycosylated hemoglobin levels compared to those receiving medical management [19]. 

Despite its numerous benefits, there is still poor awareness and utilization of metabolic surgery in Nigeria, as a recent presentation at a Bariatric and Metabolic Surgeons Society of Nigeria (BMSSN) meeting reported that 214 bariatric procedures were performed in the country in 2023. Several factors were identified, including inaccurate diagnosis of obesity, negative societal attitudes toward MBS, inadequate referral practices by other healthcare providers, the cultural association of obesity with affluence, and a dearth of knowledge regarding available treatment options among both patients and healthcare professionals [5].

Some of these themes were substantiated by our study, as 94.6% of participants were unaware of the availability of bariatric surgical procedures at their current hospitals, only 37.9% indicated knowledge of such procedures being performed within Nigeria, and almost half (47.3%) saying that they think undergoing MBS comes with a stigma in Nigeria. 

The poor uptake of MBS in Nigeria is reflective of other sub-Saharan countries. For example, in their 2022 paper, Bang et al. stated that theirs was the only unit practicing MBS in the entire country of Cameroon, with a population of about 27 million people at the time of publication [20,21]. A cursory review of the African literature reveals the majority of bariatric work being done in northern African countries and South Africa [22].

Given the poor awareness of bariatric interventions in Nigeria, doctors are likely to be the first point of contact for any patients seeking verifiable information about weight loss surgery. Therefore, they should be properly equipped with the right information during their training. Furthermore, patients meeting the criteria for surgery need to be referred to by their primary care physician. Only 2% of our respondents said they had been given sufficient information about MBS in medical school. This is similar to a study done among Polish medical students that reported 8.54% of respondents saying they had received sufficient information on MBS during their studies [13]. Fortunately, just over half the people we surveyed (55.7%) agree or strongly agree that MBS is useful for weight loss; however, a third (33.5%) are convinced of its safety. A similar study conducted in Saudi Arabia among final-year medical students and newly qualified doctors reported that 45.3% of respondents considered MBS a useful tool, while 18% viewed it as a non-safe option [12]. A potential reason for the disparity in perceived safety in our study might be because of the less exposure to bariatric procedures in the Nigerian environment.

Increasing awareness and utilization of bariatric procedures in Nigeria would require a multifaceted approach, ensuring proper education of doctors on recognizing obesity and understanding the benefits of MBS as a treatment method. This would drive increased demand for MBS and, hopefully, stimulate the development of strong multidisciplinary teams, including psychologists, dietitians, and nutritionists, improve surgeon training, and foster the development of a sustainable healthcare ecosystem [5].

One limitation of our study is the confinement of study respondents to Lagos state and the associated bias inherent with that. Lagos has one of the highest concentrations of medical healthcare workers in Nigeria and as such a sizable proportion of the country’s healthcare infrastructure is located there ​[23]​. It is thus expected that the knowledge and exposure to MBS may be lower if a more nationally representative sample was examined. Our study also faces the challenge of recall bias regarding the content of MBS in medical school curricula.

MBS remains an emerging field in Nigeria that demands comprehensive research expansion. Future research efforts should encompass multiple critical domains: exploring public attitudes and perceptions toward bariatric surgical interventions, mapping patterns of procedures carried out, analyzing training pathways for surgeons, and conducting longitudinal patient follow-up studies. These research directions will be crucial for evaluating the effectiveness of various surgical techniques in achieving weight loss and resolving obesity-related comorbidities, with comparative analyses against established Western surgical protocols and outcomes.

## Conclusions

Nigeria is confronting a growing obesity epidemic that underscores the urgent need for a comprehensive national strategy. Central to this approach is the enhancement of medical education on obesity and MBS through targeted curriculum updates, ongoing professional development programs, and specialized training workshops. Such measures are essential to improving patient care and addressing the escalating burden of obesity in Nigeria.

## Appendices

Questionnaire

Demographics

Sex a. Female b. Male

Professional level a. Medical student b. House officer

What medical school did you/are you currently attending? a. College of Medicine, University of Lagos (CMUL) b. Lagos State University College of Medicine (LASUCOM) c. Other:

For House officers, please specify your institution a. Lagos University Teaching Hospital (LUTH) b. Lagos State University Teaching Hospital (LASUTH) c. Lagos State Health Service Commission (GH Lagos, LIMH, MSCH) d. N/A (I'm a medical student)

Knowledge

The branch of medicine concerned with treatment of obesity is called a. Balneology b. Lipology c. Metabolic medicine d. Orology

Which of the following is an indication for the surgical treatment of obesity? Check all that apply.

- The presence of comorbid conditions of obesity and body mass twice higher than normal

-Patients with BMI >40 kg/m²

-The presence of comorbid conditions of obesity while BMI is >35 kg/m²

-The presence of comorbid conditions of obesity while body weight is 40 kg higher than ideal

-Failure of conservative and/or medical options of weight loss

Surgical treatment of obesity consists of a. Limiting ability to consume or digest and absorb nutrition through surgical operations on the stomach and intestines b. Removal of excess intra-abdominal fat, from the greater omentum in particular c. Removal of excess subcutaneous fat within the abdomen and lower limbs

Which of the following techniques of surgery for the treatment of obesity is considered the safest in the light of current medical knowledge? a. Classic laparotomy - wide opening of the abdominal cavity, enabling an open view of the abdominal cavity b. Laparoscopic surgery: a minimally invasive technique, also known as keyhole surgery c. Endoscopic surgery: allowing for access to the abdomen through natural orifices of the body, such as the mouth, anus, or vagina d. None of the above answers is correct

Which surgical methods for obesity management are you aware of? Check all that apply.

- Modification of hunger centers in the brain

- Sleeve gastrectomy

- Gastric by-pass

- Gastric banding

- Endoscopic introduction of a gastric balloon

- Gastric nerve excision

Which of the following are common complications of surgical management of obesity? Check all that apply.

- Malabsorption of nutrients

- Necrotizing fascitis

- Anastomotic leaks

- Increased incidence of dyslipidemia

- Hypertension

- Fat necrosis

Please evaluate the amount of information on metabolic surgery taught throughout the course of your undergraduate degree on a scale of 1-5. No Mention 1 2 3 4 5 Sufficient information taught

Do you know of metabolic bariatric surgery procedures performed at your current university hospital? a. Yes b. No

Do you know if metabolic bariatric surgeries are performed in Nigeria? a. Yes b. No c. Don’t Know

Attitude

Do you think the risk of bariatric procedures is greater than other more commonly performed abdominal surgical procedures? a. Yes b. No c. Don’t Know

Do you think there is/will be a social stigma around those who lose weight with metabolic bariatric surgery in Nigeria? a. Yes b. No c. Don’t know

I feel metabolic bariatric surgery is a useful tool for treating obesity Strongly disagree 1 2 3 4 5 Strongly agree

I feel metabolic bariatric surgery is a safe option for treating obesity Strongly disagree 1 2 3 4 5 Strongly agree

If a patient meets the standard criteria for metabolic bariatric surgery, I would recommend evaluation by a bariatric surgeon Very unlikely 1 2 3 4 5 Very Likely

If a patient asks me about surgical options for weight loss, I feel confident to provide a satisfactory answer to them Strongly disagree 1 2 3 4 5 Strongly agree
